# Breakthrough infection evokes the nasopharyngeal innate immune responses established by SARS-CoV-2–inactivated vaccine

**DOI:** 10.3389/fimmu.2023.1181121

**Published:** 2023-06-29

**Authors:** Xiaomeng He, Yingyin Cao, Yanmei Lu, Furong Qi, Haiyan Wang, Xuejiao Liao, Gang Xu, Biao Yang, Junhua Ma, Dapeng Li, Xian Tang, Zheng Zhang

**Affiliations:** ^1^ Institute of Hepatology, National Clinical Research Center for Infectious Disease, Shenzhen Third People’s Hospital, The Second Affiliated Hospital, School of Medicine, Southern University of Science and Technology, Shenzhen, Guangdong, China; ^2^ Shenzhen Research Center for Communicable Disease Diagnosis and Treatment of Chinese Academy of Medical Science, Shenzhen, Guangdong, China

**Keywords:** SARS-CoV-2, breakthrough infection, mucosal immunity, single-cell RNA sequencing, innate immune memory

## Abstract

Nasopharyngeal immune responses are vital for defense against SARS-CoV-2 infection. Although vaccination *via* muscle immunization has shown a high efficacy in reducing severity and death in COVID-19 infection, breakthrough infection frequently happens because of mutant variants and incompletely established mucosal immunity, especially in the upper respiratory tract. Here, we performed a single-cell RNA and T-cell receptor repertoire sequencing and delineated a high-resolution transcriptome landscape of nasopharyngeal mucosal immune and epithelial cells in vaccinated persons with breakthrough infection and non-vaccinated persons with natural infection as control. The epithelial cells showed anti-virus gene expression diversity and potentially recruited innate immune cells into the nasopharyngeal mucous of vaccinated patients. Upon infection, they released significant pro-inflammatory cytokines and chemokines by macrophages and monocytes and expressed antigen-presenting relevant genes by dendritic cells. Such immune responses of nasopharyngeal innate immune cells would facilitate the strengthened expression of cytotoxic genes in virus-specific T-cell or B-cell differentiation into antibody-secreting cells at the early stage of breakthrough infection through cell interaction between innate and adaptive immune cells. Notably, these alterations of nasopharyngeal immune cells in breakthrough infection depended on the activated Nuclear factor-κB (NF-κB) and NOD-, LRR- and pyrin domain-containing protein 3 (NLRP3) signaling rather than type I interferon responses due to the general reduction in interferon-stimulated gene expression. Our findings suggest that vaccination potentially strengthens innate immune barriers and virus-specific memory immune cell responses, which could be quickly activated to defend against variant breakthrough infection and maintain nasopharyngeal epithelial cell integrity. Thus, this study highlights the necessity of a boost *via* nasal mucous after intramuscular immunization.

## Introduction

The worldwide health threat, coronavirus disease 2019 (COVID-19), caused by severe acute respiratory syndrome coronavirus 2 (SARS-CoV-2), has led to more than 600 million infections and 6 million deaths (https://coronavirus.jhu.edu/map.html). Despite the effectiveness of anti-viral drugs in preventing severe disease and reducing mortality, promoting and popularizing vaccines are the most vital ways to impede virus spread (1, 2). Similar to recovered patients with COVID-19, vaccinated subjects (including those vaccinated with mRNA and inactivated SARS-CoV-2 vaccines) displayed an efficient induction of virus-specific T-cell responses and neutralizing antibodies among a wide range of tissues, such as peripheral blood, lymph nodes, and upper respiratory tract mucous (3–10). This might rationally explain the restrained virus replication, shedding, tissue localization (11), and even the clinically reported decline of severe incidence and mortality after vaccination (4, 12–14). Meanwhile, vaccination elicited the milder innate immune responses than SARS-CoV-2 natural infection, indicated by the slight accretion of monocyte ratio in peripheral blood, demonstrating the safety of vaccines (15, 16).

Although the efficacy and safety of the vaccine have been demonstrated, some breakthrough infections were caused by reduced neutralizing antibodies due to virus mutants (17–21). A great deal of investigation on the immune landscape following vaccination emphasized their similarity to mild disease, including the small-scaled reduction of lymphocytes and dendritic cells ratio, the slight expression of type I interferon (IFN)–stimulated genes (ISGs), and inflammation-related genes (7, 16, 22, 23). Nonetheless, the distinction in anti-viral immune responses between breakthrough and natural infections is largely unknown. Breakthrough infection, to some extent, mimics mucosal booster vaccination. In addition, although inactivated vaccines showed similarity in inducing peripheral humoral and adaptive cell immune responses with mRNA vaccines, less evidence about establishment of local mucosal virus-specific adaptive immunity after immunization with inactivated vaccines was indicated. Therefore, understanding local mucosal immune responses in the upper respiratory tract especially in breakthrough infections is important for designing mucosal vaccines.

This study compared the nasopharyngeal immune landscape between breakthrough and natural infections *via* single-cell transcriptome combined T-cell receptor (TCR) (VDJ) repertoire sequencing. We found that the strengthened interaction between nasopharyngeal epithelial cells and macrophages from vaccine breakthrough infections in a chemokine receptor–dependent manner elevated the pro-inflammatory, chemotactic, and antigen-presenting–related gene expression, respectively, in macrophages, monocytes, and dendritic cells, thereby promoting the activation and clonal expansion of vaccine-established virus-specific T cells and boosting the cytotoxic role of CD8^+^ T effector memory cells in upper respiratory tract mucous during SARS-CoV-2 re-infection. In addition, we observed that the enhanced function of innate and adaptive immune cells did not rely on the responses to type I IFN due to the general decline of ISG expression from vaccinated patients and might be interrelated to the restrained virus replication achieved by established virus-specific adaptive memory responses. Conversely, vaccination promoted squamous, secretory, and ciliated cells transcribed from a wider range of genes, including inflammation and ISGs and reactive oxygen species (ROS) and protein translation–related genes, which are crucial for anti-viral responses.

These findings suggest that the upper respiratory tract immune responses are largely remodeled vaccine breakthrough infections. First, vaccination successfully establishes mucous resident memory T cells and influences innate and adaptive immune responses during virus breakthrough infection. Second, the more kinds of anti-viral strategies are adopted by epithelial cells in addition to ISGs. Finally, inactivated SARS-CoV-2 vaccines are beneficial for maintaining the integrated mucosal structure by modulating nasopharyngeal immune responses. Our work first displayed the nasopharyngeal transcriptome landscape in SARS-CoV-2–inactivated vaccine–immunized subjects with breakthrough infection and revealed the potential advantage of subsequent immunization with intranasal SARS-CoV-2 vaccine after intramuscular immunization at the cellular and molecular levels.

## Materials and methods

### Design

All nasopharyngeal swabs were collected from healthy donors (n = 9) and patients with COVID-19 (n = 29) hospitalized in Shenzhen Third People’s Hospital. Healthy donors were divided into two groups, HC-V (n = 3) and HC-NV (n = 6), according to whether they received vaccines for SARS-CoV-2. Patients who received one or two doses of inactivated vaccine for SARS-CoV-2 were noted as COVID-V (n = 9). In addition, patients who received no vaccine (COVID-NV, n = 22) were grouped as Alpha (infected with SARS-CoV-2 Alpha variant, n = 7), Delta (infected with Delta variant, n = 5), and WT (infected with wild-type variant, n = 10). All COVID-V patients were infected with the Alpha variant. Detailed information on healthy volunteers and patients is provided in **Supplementary Table S1**. The nasopharyngeal swabs from patients for single-cell RNA sequencing (scRNA-seq) were collected between 0 and 10 days after confirming SARS-CoV-2 infection *via* PCR. For vaccinated healthy volunteers, nasopharyngeal swabs for scRNA-seq were collected 14 days after second dose vaccination. Participants in infected groups included asymptomatic, mild, moderate, and severe individuals, categorized according to the “Diagnosis and Treatment Protocol of COVID-19 (the 7th Tentative Version)” by the National Health Commission of China (http://www.nhc.gov.cn/yzygj/s7653p/202003/46c9294a7dfe4cef80dc7f5912eb1989.shtml). All participants provided a written informed consent for sample collection and subsequent analyses.

This study was conducted according to the ethical principles of the Declaration of Helsinki. Ethical approval was obtained from the Research Ethics Committee of Shenzhen Third People’s Hospital (2022003).

### Sample collection

Nasopharyngeal samples were collected by a trained healthcare provider using FLOQSwabs (Copan flocked swabs) following the manufacturer’s instructions. Collectors would don personal protective equipment, including a gown, non-sterile gloves, a protective N95 mask, a bouffant, and a face shield. The patient’s head was tilted back slightly, and the swab was inserted along the nasal septum, above the floor of the nasal passage to the nasopharynx, until slight resistance was felt. The swab was then left in place for several seconds to absorb secretions and slowly removed while rotating the swab. The swab was then placed into a tube containing 5 ml of RPMI 1640 medium with 5% heat-inactivated fetal bovine serum (FBS) for subsequent isolation of cells.

### Single-cell collection from nasopharyngeal swabs

Nasopharyngeal swabs were incubated in 5 ml of RPMI 1640 medium (Hyclone Laboratories Inc., Omaha, NE, USA) with 5% heat-inactivated FBS (Gibco) for 15 min with agitation. Then, 5 ml of RPMI from the swab incubation was centrifuged at 1,500 rpm for 5 min at 4°C to pellet cells, the supernatant was discarded, and the cell pellet was resuspended in 1 ml of RPMI with 5% FBS. Nasopharyngeal swabs were placed in a new medium for another incubation period of 5 min with agitation. The medium from the second swab incubation combined with cell suspension was centrifuged at 1,500 rpm for 5 min at 4°C. The cell pellet was washed twice in Dulbecco’s phosphate-buffered saline (DPBS) (Thermo Fisher Scientific, Waltham, MA, USA) with 2% FBS, and resuspended cells were filtered using a 70-mm nylon strainer (BD Falcon). Cells were diluted to 2,000 cells/µl for scRNA-seq. If cell concentration was less than 2,000 cells/µl, then all cells were input into scRNA-seq.

### 5′ VDJ-integrated scRNA-seq library construction

The cell suspension was loaded onto a chromium single-cell controller (10X Genomics, Pleasanton, CA, USA) to generate single-cell gel beads in the emulsion (GEMs) according to the manufacturer’s protocol. Reverse transcription occurred inside each GEM, after which complementary DNA (cDNA) were pooled together for amplification and library construction. The resulting library products consisted of Illumina adapters and sample indices, allowing the pooling and sequencing of multiple libraries on the next-generation short-read sequencer.

### Single-cell RNA-seq data processing

The raw 5′ scRNA-seq reads were mapped against the human reference genome (GRCh38) using cellranger (v6.1.1; 10X Genomics). The generated feature-barcode count matrices were filtered according to the following criterion: 1) cells expressing ≤ 100 genes, ≥ 6,000 genes, and ≥ 25% mitochondrial transcripts were discarded; 2) genes that were expressed in ≤3 cells were deleted. The filtered feature-barcode count matrices were loaded into R (v4.0.2) with Seurat package (v4.0.4) (PMID: 34062119) to perform batch effect correction, cell clustering, and dimension reduction. Specifically, the gene expression matrices were normalized on the basis of the total read count and log-transformed using the “NormalizeData” function. To correct batch effects, the samples were aligned using the “IntegrateData” function with k.weight = 10 using the canonical correlation analysis after choosing the top 2,000 highly variable genes in each sample. Principal component analysis (PCA) was conducted using the “RunPCA” function, followed by constructing a shared nearest neighbor graph using the “FindNeighbors” function and cell clustering using the “FindClusters” function with a resolution of 0.4. The cell clusters were finally visualized *via* uniform manifold approximation (UMAP) using the top 20 principal components.

### Cell re-clustering

Epithelial cells, immune cells, and T cells were separately re-clustered using the Seurat pipeline as described above. Unlike whole cell population clustering, cell re-clustering was performed using the “FindClusters” function with a resolution of 0.8.

### Cell-type annotation through canonical markers

We calculated markers expressed in each cell cluster using the “FindAllMarkers” function. The cell clusters were manually annotated *via* canonical markers specifically expressed on these cells [average logarithm two-fold change (log2FC) > 1, p_val_adj < 0.05].

### Identification of SARS-CoV-2–infected cells in patients with COVID-19

The SARS-CoV-2 genome (Refseq-ID: NC045512) was added as an additional chromosome to the human reference genome (GRCh38). Moreover, we added an entry summarizing the entire SARS-CoV-2 genome as a “gene” to the GRCh38 annotation gtf file. The genome was indexed using “cellranger mkref”. We then used cellranger (v6.1.1; 10X Genomics) to map the sequenced reads against the reconstructed reference. The sequenced reads mapped against the SARS-CoV-2 genome in a cell were identified as SARS-CoV-2–infected cells.

### Single-cell RNA-seq signature score

IFN-responsive score, cytokine score, chemokine score, and PNA (pyroptosis, necrosis, and apoptosis) score were calculated using the “AddModuleScore” function implemented in the Seurat package. IFN-responsive score was calculated using the following genes: *IFITM1*, *OAS1*, *OAS2*, *OAS3*, *IFIT1*, *IFIT2*, *IFIT3*, *IFI6*, *IFI27*, *STAT1*, *STAT2*, *IFI44*, *ISG15*, *ISG20*, *MX1*, *IRF1*, *IRF9*, *APOBEC3C*, *GBP5*, *IFNGR1*, *IFNGR2*, *IFNAR1*, and *IFNAR2*. Cytokine score was calculated using genes, including *IFNA1*, *IFNA10*, *IFNA13*, *IFNA14*, *IFNA16*, *IFNA17*, *IFNA2*, *IFNA21*, *IFNA4*, *IFNA5*, *IFNA6*, *IFNA7*, *IFNA8*, *IFNE*, *IFNG*, *IFNK*, *IFNW1*, *IFNL1*, *IFNL2*, *IFNL3*, *IL1A*, *IL1B*, *IL2*, *IL3*, *IL4*, *IL5*, *IL6*, *IL7*, *IL8*, *IL9*, *IL10*, *IL12*, *IL13*, *IL15*, *IL17*, *IL18*, *IL21*, *IL22*, *IL23*, and *IL33*. Chemokine score was calculated using *CCL1*, *CCL2*, *CCL3*, *CCL5*, *CCL7*, *CCL8*, *CCL11*, *CCL13*, *CCL3*, *CCL15*, *CCL17*, *CCL21*, *CCL22*, *CCL24*, *CCL26*, *CCL27*, *CXCL1*, *CXCL5*, *CXCL8*, *CXCL9*, *CXCL10*, *CXCL11*, *CXCL13*, *TNF*, *LTA*, *EGF*, *VEGFA*, *PDGFA*, *PDGFB*, *TGFB1*, *FGF2*, *TPO*, *TNFSF10*, *CSF1*, *CX3CL1*, *FLT3LG*, *TSLP*, and *KITLG*. The genes not detected in a specific cell type were excluded when performing signature score analysis in that cell type.

### Single-cell TCR-seq data processing

The amino acid and nucleotide sequence of TCR chains were assembled and annotated *via* the “cellranger vdj” function in CellRanger (version 6.1.1). Only cells with paired TCRα and TCRβ chains were included in the clonotype analysis; cells sharing the same TCRα- and TCRβ-CDR3 amino acid sequences were assigned to the same TCR clonotype.

### Analysis of DEGs

The “FindMarkers” function in Seurat with the MAST algorithm was used to analyze differentially expressed genes (DEGs). For each pairwise comparison, we run the “FindMarkers” function with parameters of test.use = “MAST”. Genes were defined as significantly upregulated if there was log2FC > 1 and adjusted P < 0.01. The genes with log2FC < −1 and adjusted P < 0.01 were considered significantly downregulated. The enrichment analysis of significantly upregulated and downregulated genes was conducted using clusterProfiler (v4.2.2) (PMID: 34557778) in R. Only the gene ontology (GO) term of biological process was displayed.

### Statistics and data visualization

The Student’s t-test (t.test in R, two-sided, unadjusted for multiple comparisons) was used for pairwise comparisons of the cell proportions between different groups. In addition, the Mann–Whitney U-test (wilcox.test in R, two-sided, nonparametric test) was used to compare scores of the functional genes between different groups. Box, bar, and point plots were visualized using the ggplot2 package (v3.3.3).

## Results

### Breakthrough infection potentially generated memory humoral immune responses

**Figure 1:**
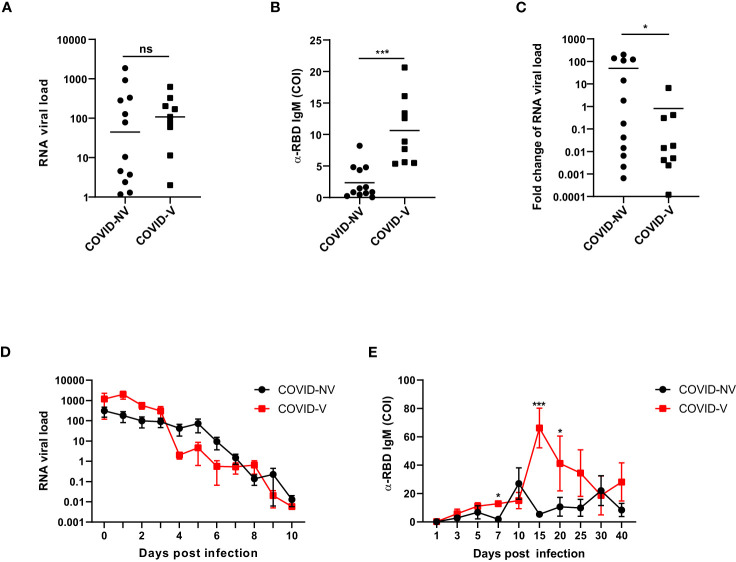
Breakthrough infection potentially generated memory humoral immune responses. **(A)** The RNA viral load of nasopharyngeal swabs from vaccinated and non-vaccinated patients with COVID-19 at the first day after proven virus positive by qPCR measurement of SARS-CoV-2 nucleoprotein gene (COVID-NV, n = 12, seven individuals infected with Alpha variant and five infected with Delta variant; COVID-V, n = 9, nine infected with Alpha variant). **(B)** The amount of serum anti-RBD IgM in vaccinated and non-vaccinated patients with COVID-19, 4 days after tested positive for SARS-CoV-2 (COVID-NV, n = 12; COVID-V, n = 9). **(C)** Fold change of SARS-CoV-2 viral load of nasopharyngeal swabs from vaccinated and non-vaccinated patients with COVID-19, 4 days after tested positive for SARS-CoV-2, which calculated by normalizing RNA viral load from nasopharyngeal swabs tested 4 days after PCR-proven virus infection to RNA viral load at the first day after proven virus positive (COVID-NV, n = 12; COVID-V, n = 9). **(D)** The RNA viral load of nasopharyngeal swabs from vaccinated and non-vaccinated patients with COVID-19 at different time points (COVID-NV, n = 12; COVID-V, n = 9). **(E)** The concentration of serum anti-RBD IgM in vaccinated and non-vaccinated patients with COVID-19 at different time points (COVID-NV, n = 12; COVID-V, n = 9). Data from **(A–C)** were analyzed by Student’s t-test, and data from **(D, E)** were analyzed by two-way ANOVA; *P <0.05, and ***P < 0.001; ns, not significant.

We first investigated the clinical parameters among the donors with breakthrough infection and natural infection. The nasopharyngeal SARS-CoV-2 viral load was comparable at the first day after proven positive for SARS-CoV-2 infection between breakthrough infection and natural infections (**Figure 1A**). However, the serum RBD (receptor-binding domain)–specific antibody was initialized earlier and was higher in patients with breakthrough infection than in those with natural infections, as early as 4 days after testing positive for SARS-CoV-2 (**Figure 1B**). The viral load was rapidly reduced, accompanied by the generation of virus-specific IgM at 4 days after infection (**Figure 1C**). Because both vaccinated and non-vaccinated hospitalized individuals were medically treated, they had similar virus clearance times, peak titers of RBD-specific antibody, inflammatory cytokine levels, and hospitalization times (**Figures 1D, E**; **Figures S1A–G**). These clinical data indicated that inactivated SARS-CoV-2 vaccination potentially set up the memory humoral immune responses.

### scRNA-seq characterized the nasopharyngeal immune environment and revealed the epithelium integrity maintained by vaccination with breakthrough infection

**Figure 2:**
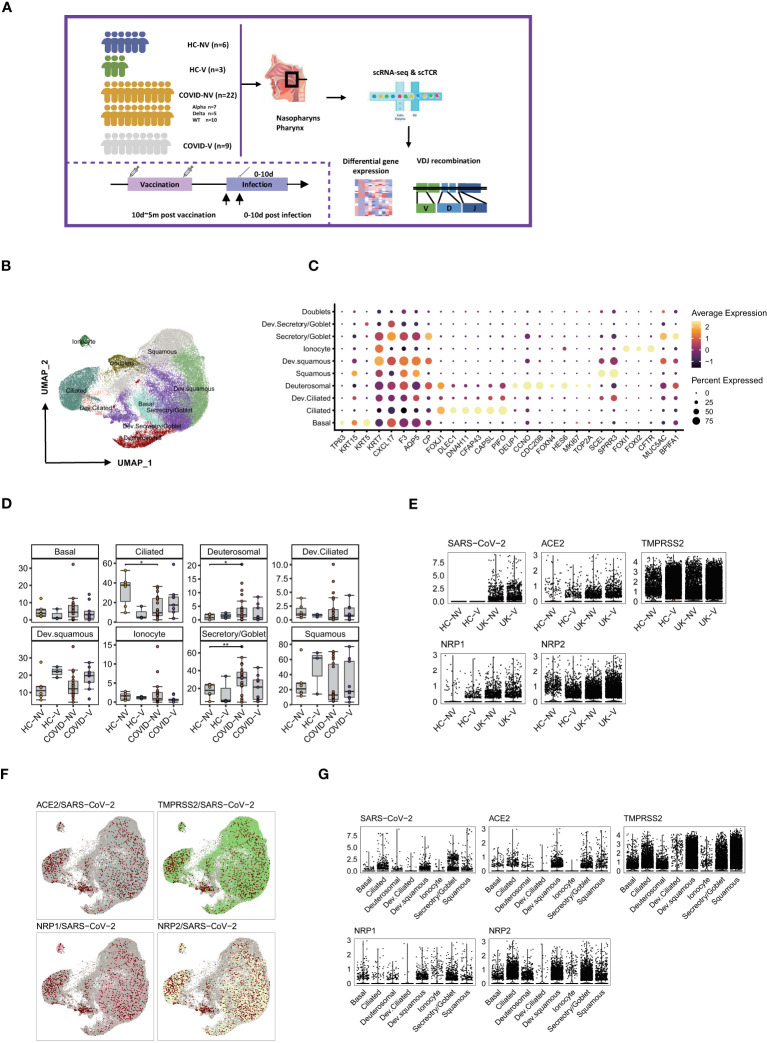
Overall characteristics of nasopharyngeal epithelial cells in SARS-CoV-2 vaccine breakthrough infections. **(A)** Graphical overview of the study. Nasopharyngeal swabs from six healthy volunteers (HC-NV), three vaccinated healthy participants (HC-V), 22 non-vaccinated patients with COVID-19 (COVID-NV, comprised of seven Alpha variant infections, five Delta variant infections, and 10 WT infections) and nine vaccine breakthrough infections (COVID-V, Alpha variant infections) were collected, for scRNA-seq 0–10 days after PCR-proven SARS-CoV-2 infection. **(B)** UMAP representation of the 10 epithelial cell types from the integrated nasopharyngeal scRNA-seq dataset. **(C)** Canonical gene expression markers for nasopharyngeal epithelial cells clustering. **(D)** Proportions of various nasopharyngeal epithelial cells from vaccinated and non-vaccinated healthy participants and patients with COVID-19. **(E)** Normalized abundance of SARS-CoV-2 and receptors for SARS-CoV-2 cell entry from vaccinated and non-vaccinated healthy participants and patients with COVID-19. **(F)** UMAP of distribution of SARS-CoV-2 RNA and receptors for SARS-CoV-2 cell entry among various nasopharyngeal epithelial cell clusters. **(G)** Normalized abundance of SARS-CoV-2 and receptors for SARS-CoV-2 cell entry from different nasopharyngeal epithelial cell types. HC-NV, n = 6; HC-V, n = 3; COVID-NV, n = 22; COVID-V, n = 9. Data from different groups in **(D)** and were analyzed by two-sided Student’s t-test; *P <0.05, **P < 0.01.

Regarding peripheral immune responses, nasopharyngeal immune responses may directly determine the risk of virus transmission into the lower respiratory airway. Hence, we collected the nasopharyngeal swabs from patients with or without SARS-CoV-2 vaccination within 10 days after PCR-proven SARS-CoV-2 infection and investigated the nasopharyngeal mucosal immune landscape of both groups. Sequencing showed that patients with breakthrough infection were infected with SARS-CoV-2 alpha variants (B.1.1.7). They had received single or two doses of inactivated SARS-CoV-2 vaccine (CoronaVac or Sinopharm BIBP) 10 days to 5 months before infection. Meanwhile, the non-vaccinated natural infections comprised patients infected with WT, Alpha, or Delta variants. In addition, the nasopharyngeal swabs from healthy volunteers who received inactivated SARS-CoV-2 vaccine or not were collected as controls. We performed the droplet-based single-cell sequencing (10X Genomics) to profile T-cell VDJ repertoires integrated with 5′ gene expression for the epithelial and immune cells from nasopharyngeal swabs (**Figure 2A**; **Supplementary Table S1**).

On the basis of the integrated single-cell gene expression profiles, we recovered the transcriptomes of 108:103 high-quality cells from 24 samples, which were subsequently combined and projected in a 2D space using the UMAP. Unsupervised clustering analyses and marker mapping enabled us to uncover 12 major types, including six epithelial and six immune cell types (**Figure S2A**) based on the specific cell lineage markers (**Figure S2C**). The cell fraction of the 12 major cell types is shown in **Figures S2B, D**.

Because the epithelial cells comprise 60%~80% of the nasopharyngeal mucous, we first clustered the epithelial cells and analyzed their transcriptional alteration between vaccinated and non-vaccinated patients. According to the expression level of specific markers for nasopharyngeal epithelial cells, such as keratin 5 (KRT5), mucin 5AC (MUC5A), BPI fold containing family A member 1 (BPIFA1), forkhead box J1 (FOXJ1), zinc finger protein 185 with LIM domain (SCELL), forkhead box I1 (FOXI1) and deuterosome assembly protein 1 (DEUP1), the epithelial cells were classified into six predominant sub-clusters: basal cells, goblet/secretory cells, ciliated cells, squamous cells, deuterosomal cells, and ionocytes; three intermediate sub-clusters: developing goblet/secretory cells (expressing markers of both goblet/secretory cells and basal cells), developing squamous cells (expressing markers of both squamous cells and goblet/secretory), and developing ciliated cells (expressing markers of both ciliated cells and squamous cells); as well as a group of doublets that would be precluded in further analysis (24, 25) (**Figures 2B, C**). Three intermediate sub-populations were positioned in the middle of two cell stages at the epithelial-differentiating trajectory, which may originate from basal cells, then progress through goblet/secretory cells into squamous cells, and terminally develop into ciliated cells. On the basis of the proportions of epithelial subsets, nasopharyngeal mucous was mainly characterized by ciliated cell loss and goblet/secretory cell compensated propagation after SARS-CoV-2 infection (**Figure 2D**), which was consistent with previous reports (24). In addition, infection with various variants had less effect on the consistency of nasopharyngeal mucous cells (**Figure S3A**). However, breakthrough infection did not lead to ciliated cell loss and the compensated proliferation of goblet/secretory cells to some extent, indicating that vaccination potentially maintained the nasopharyngeal epithelium integrity. Notably, squamous cells and developing squamous cells propagated in vaccinated persons, including patients with breakthrough infection and healthy individuals, inferring the most intimate involvement of squamous cells during infection (**Figure 2D**).

We assessed the impact of vaccination on virus entry by detecting the genes associated with virus infection and distribution. The dominant receptor angiotensin converting enzyme 2 (ACE2) and auxiliary receptors transmembrane serine protease 2 (TMPRSS2), neuropilin 1 (NRP1) and neuropilin 2 (NRP2) were comparably expressed in different groups regardless of virus variants and vaccination (**Figure 2E**). Therefore, these receptors for virus entry seemed to be constitutively expressed on the epithelium of the upper respiratory tract. Although the non-vaccinated patients with COVID-19 showed similar receptor expression patterns at the early infection stage with vaccine breakthrough infections, where only the two groups of individuals infected alpha variants were co-detected with virus RNA in nasopharyngeal epithelium (**Figure 2E**), this might be explained by the disparity of swab sample quality. The virus RNA distribution seemed highly associated with *ACE2* transcription rather than the other three auxiliary receptors. Most SARS-CoV-2 RNA was enriched in four epithelial subsets, including ciliated, goblet/secretory, squamous, and developing squamous cells, which all displayed the most abundant *ACE2* expression (**Figures 2F, G**). However, the auxiliary receptors TMPRSS2, NRP1, and NRP2 were evenly expressed by different epithelial populations (**Figure 2F**), indicating that ACE2 accounted for most of the virus entry. These data suggested a protective function of vaccination against nasopharyngeal mucous damage and maintenance of the epithelium composition.

### The anti-viral functions of nasopharyngeal epithelial cells were ameliorated in breakthrough infections *via* diverse pathways

**Figure 3:**
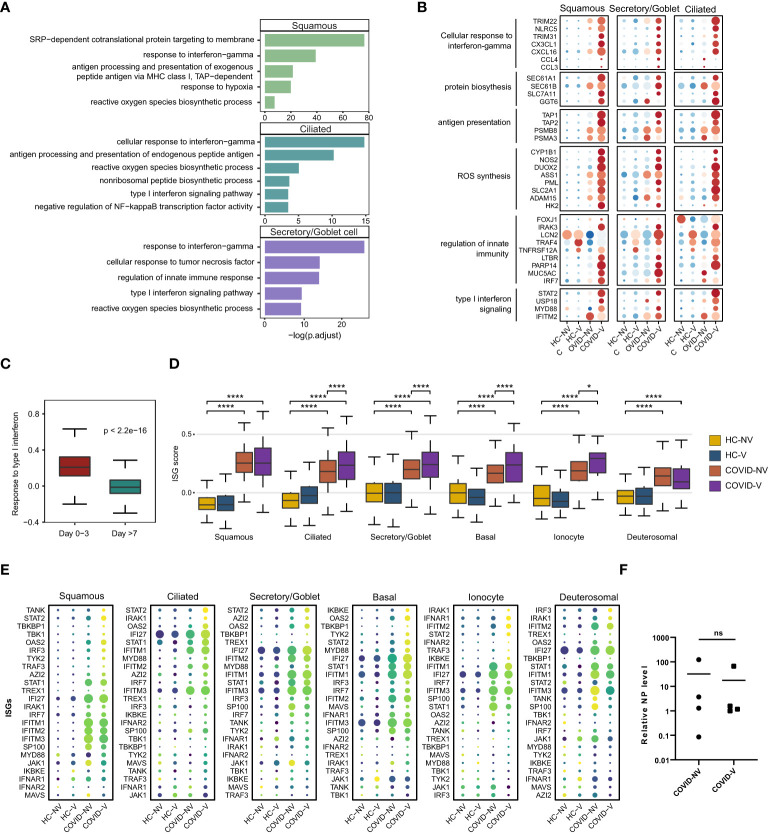
Vaccination breakthrough infections experience type I interferon signaling enhancement in nasopharyngeal epithelial cells during early stage of infection. **(A)** Enrichment of GO biological process terms for differentially expressed genes (DEGs) transcribed at higher levels in SARS-CoV-2 RNA-positive cells from vaccine breakthrough infections (COVID-V), compared with non-vaccinated natural infections (COVID-NV). **(B)** The heatmaps of selected DEGs enriched in signaling pathways actively transcribed in SARS-CoV-2 RNA-positive cells from vaccine breakthrough infections (COVID-V). **(C)** Interferon-stimulated gene (ISG) score of patients proven SARS-CoV-2 positive within 3 days (n = 8; four natural infections and four breakthrough infections) and over 7 days (n = 18, 13 natural infections and five breakthrough infections). **(D)** The gene expression score of ISG in various nasopharyngeal epithelial cell subsets from vaccinated and non-vaccinated healthy participants and patients with COVID-19, 0–3 days after infection (HC-NV, n = 6; HC-V, n = 3; COVID-NV, n = 4; COVID-V, n = 4). **(E)** The heatmaps of selected ISGs in different epithelial cells subsets from vaccinated and non-vaccinated healthy participants and patients with COVID-19, 0-3 days after infection. **(F)** The relative expression level of SARS-CoV-2 nucleoprotein gene of nasopharyngeal swabs from vaccinated and non-vaccinated patients with COVID-19 at the time point of scRNA-seq performed (COVID-NV, n = 4; COVID-V, n = 4). HC-NV, n = 6; HC-V, n = 3; COVID-NV, n = 22; COVID-V, n = 9 except indicated. Data from **(C, D)** were analyzed by Mann–Whitney U-test, and data from **(F)** were analyzed by two-side Student’s t-test; *P <0.05 and ****P <0.0001; ns, not significant.

We further analyzed the gene expression changes caused by inactivated SARS-CoV-2 vaccines during breakthrough infection. The epithelial cells with detectable SARS-CoV-2 RNA were first analyzed using the signaling pathway enriched by DEGs in participants with breakthrough and natural infections. GO analysis indicated a significant transcription diversity in a wide range of SARS-CoV-2–infected epithelial cells from patients with breakthrough infection. Many classic inflammation- and anti-viral–related genes, such as type I IFN signaling pathway, response to IFN-gamma, innate immune response regulation, antigen presentation, ROS, and protein translation, which have been demonstrated effective in anti-viral responses (26–28), were generally upregulated in the infected ciliated, goblet/secretory, and squamous cells from vaccinated patients with breakthrough infection (**Figures 3A, B**).

Notably, the type I IFN responses by respiratory airway epithelial cells were increasingly activated as early as within 3 days after SARS-CoV-2 RNA-positive detection and subsequently dramatically dropped during 4–7 days after infection regardless of vaccination (**Figure 3C**). We further analyzed the early type I IFN responses within 3 days after infection (29, 30). It is noteworthy that ISG burst induced by SARS-CoV-2 infection was increased in nearly all types of epithelial cells in vaccinated patients (**Figures 3D, E**); this phenomenon did not result from the different nasopharyngeal viral load when scRNA-seq was performed between two groups of participants or the individual variation (**Figure 3F**, **S3B**). These data indicated that the ISG responses combined with diverse anti-virus strategies, including ROS synthesis and protein translation, were significantly increased in breakthrough infection compared to natural infection, suggesting that vaccination could enhance the anti-virus functions of nasopharyngeal epithelial cells against the early virus infection.

### SARS-CoV-2 vaccination increased the pro-inflammatory and chemotactic function of nasopharyngeal myeloid cells during SARS-CoV-2 breakthrough infection

**Figure 4:**
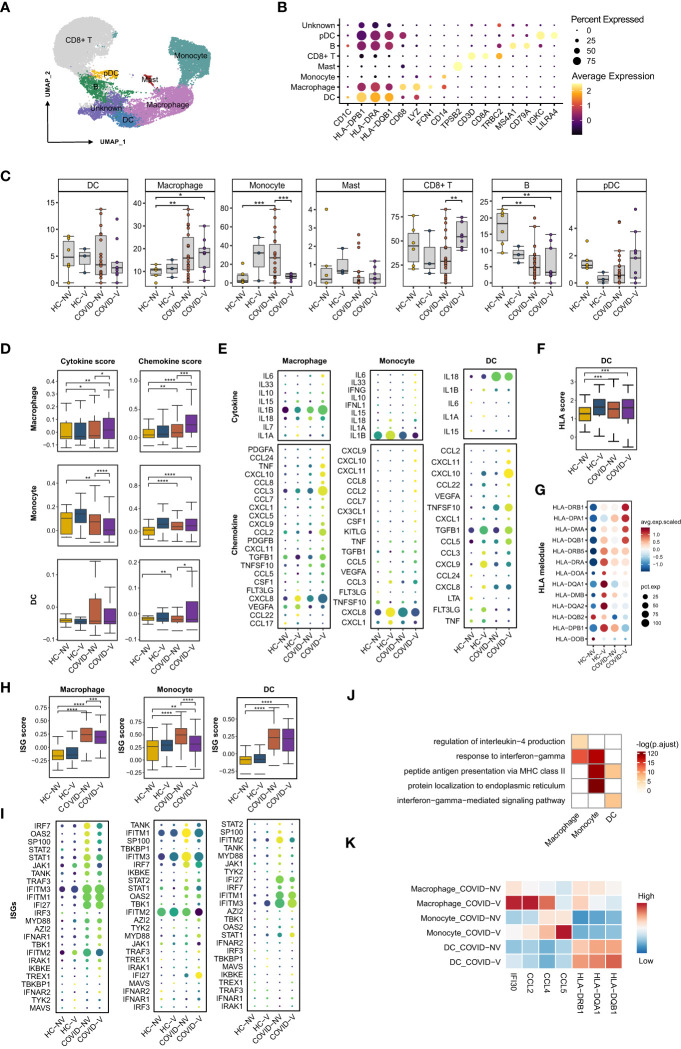
SARS-CoV-2 vaccination evidently enhances pro-inflammatory function of nasopharyngeal innate immune cells while restrains ISG expression. **(A)** UMAP representation of the eight immune cell types from the integrated nasopharyngeal scRNA-seq dataset. **(B)** Canonical gene expression markers for nasopharyngeal immune cells clustering. **(C)** Proportions of various nasopharyngeal immune cells from vaccinated and non-vaccinated healthy participants and patients with COVID-19. **(D)** The gene expression score of cytokine and chemokine in macrophages, monocytes, and dendritic cells from vaccinated and non-vaccinated healthy participants and patients with COVID-19. **(E)** The heatmaps of cytokines and chemokines in monocyte and dendritic cell from vaccinated and non-vaccinated healthy participants and patients with COVID-19. **(F)** The gene expression score of MHC class II molecules in dendritic cells from vaccinated and non-vaccinated healthy participants and patients with COVID-19. **(G)** The heatmaps of MHC class II molecules in dendritic cells from vaccinated and non-vaccinated healthy participants and patients with COVID-19. **(H)** The gene expression score of ISG in macrophages, monocytes, and dendritic cells from vaccinated and non-vaccinated healthy participants and patients with COVID-19. **(I)** The heatmaps of selected ISGs in macrophages, monocytes, and dendritic cells from vaccinated and non-vaccinated healthy participants and patients with COVID-19. **(J)** Enrichment of GO biological process terms for DEGs in macrophages, monocytes, and dendritic cells from COVID-V, compared with COVID-NV. **(K)** The representative DEGs enriched in signaling pathways specifically expressed in macrophages, monocytes, and dendritic cells from COVID-V. HC-NV, n = 6; HC-V, n = 3; COVID-NV, n = 22; COVID-V, n = 9. Data from **(C)** were analyzed by two-side student’s t-test, and data from **(D, F, H)** were analyzed by Mann–Whitney U-test; *P <0.05, **P < 0.01, ***P < 0.001 and ****P < 0.001.

Nasopharyngeal immune cells were sub-classified into CD8^+^ T cells, B cells, conventional dendritic cells (cDCs), macrophages, monocytes, mast cells, and plasmacytoid dendritic cells (pDCs), according to the main marker genes (31, 32) (**Figures 4A, B**). Like in previous studies (15), SARS-CoV-2 infection was responsible for the reduction of CD8^+^ T cells and cDCs and the accretion of macrophages, monocytes, and pDCs (**Figure 4C**), which subsequently caused the accumulation of both pro-inflammatory cytokines and ISG-producing cells and the inhibition of virus-specific cytotoxic function. Notably, vaccination with inactivated vaccines effectively impeded the alteration and maintained cell subset proportion similar to normal level except for macrophages that experienced slight accretion despite the breakthrough infection (**Figure 4C**).

We also investigated whether the function of nasopharyngeal mucosal innate immune cells was modulated in patients with breakthrough infections. The crucial function of innate immune cells, such as macrophages, monocytes, and dendritic cells, is to release pro-inflammatory cytokines and chemokines for immune cell recruitment. Vaccination boosted both pro-inflammatory and chemokine gene expression in macrophages, as well as the cytokine or chemokine genes, respectively, in monocytes and dendritic cells during breakthrough infection (**Figures 4D, E**), which is beneficial for the activation of subsequent adaptive immune responses and virus clearance at the early stage of infection. IL-6, IL-1β, and IL-33 produced by macrophages and monocytes as well as CCL2, CCL7, CCL8, and CCL22 produced by macrophages or dendritic cells would further recruit macrophages and monocytes into inflamed mucous, whereas CXCL9 and CXCL10 are responsible for attracting T cells (**Figure 4E**). In contrast to ISGs, expression of cytokines and chemokines by innate immune cells maintained at least for 10 days, even if reduced during the next week after infection (**Figures S4D, E**). In addition, the contribution to releasing pro-inflammatory cytokines and chemokines by macrophages from breakthrough infections could be more long-lasting than monocytes, indicated by the higher cytokine or chemokine scores in macrophages during both 0–3 days and over 7 days after infection, contrary to the evident accretion in monocytes during 0–3 days (**Figures S4F, G**).

In addition, as the linker of innate and adaptive immunity, the antigen-presenting function of dendritic cells also needs further investigation under vaccine breakthrough conditions. The expression of major histocompatibility complex (MHC) class II molecules was generally used to evaluate the antigen-presenting capacity for dendritic cells. We found a slight increase in the human leukocyte antigen (HLA) score in dendritic cells from breakthrough infections. Similarly, most MHC class II molecules were upregulated at the transcription level in breakthrough infections, especially HLA-DRB1, HLA-DPA1, HLA-DMA, and HLA-DQB1b (**Figures 4F, G**), indicating the enhanced antigen-presenting capacity by vaccination. Different from ISGs and cytokines, MHC II gene expression on dendritic cells could be maintained for 7 days after breakthrough infection (**Figure S4C**).

Importantly, the improved function by these innate immune cells seemed to be type I IFN-independent because ISG expression was observed to be downregulated in these nasopharyngeal macrophages, monocytes, and dendritic cells (**Figures 4H, I**; **S4B, H**), which was in contrast to epithelial cells (**Figures 4D, E**). This phenomenon was even more evident when the patients were analyzed individually (**Figure S4H**). Although infection with various SARS-CoV-2 variants facilitated the distinct extent of alteration on pro-inflammatory and antigen processing function or type I IFN responses (**Figures S4A, B**), vaccination-mediated function development and ISG response prohibition of innate immune cells could be confirmed due to the individual sample comparison (**Figures S4C, H**).

We investigated the possible genes specifically upregulated in innate immune cells from vaccine breakthrough infections. GO analysis was performed to specify the vaccination-related genes. We found a significant enrichment of inflammation-related genes in macrophages and monocytes, including cellular responses to IFN-gamma, regulation of IL-4 production, and antigen-processing–related genes such as antigen processing and presentation of exogenous peptide antigen *via* MHC class II in dendritic cells (**Figures 4J, K**). These data collectively proved the enhancement of pro-inflammation and antigen-presenting function of innate immune cells by vaccination against subsequent breakthrough infection.

### Vaccination promoted nasopharyngeal B-cell differentiation into plasma cells and strengthened CD8^+^ Tm’s cytotoxic function

**Figure 5:**
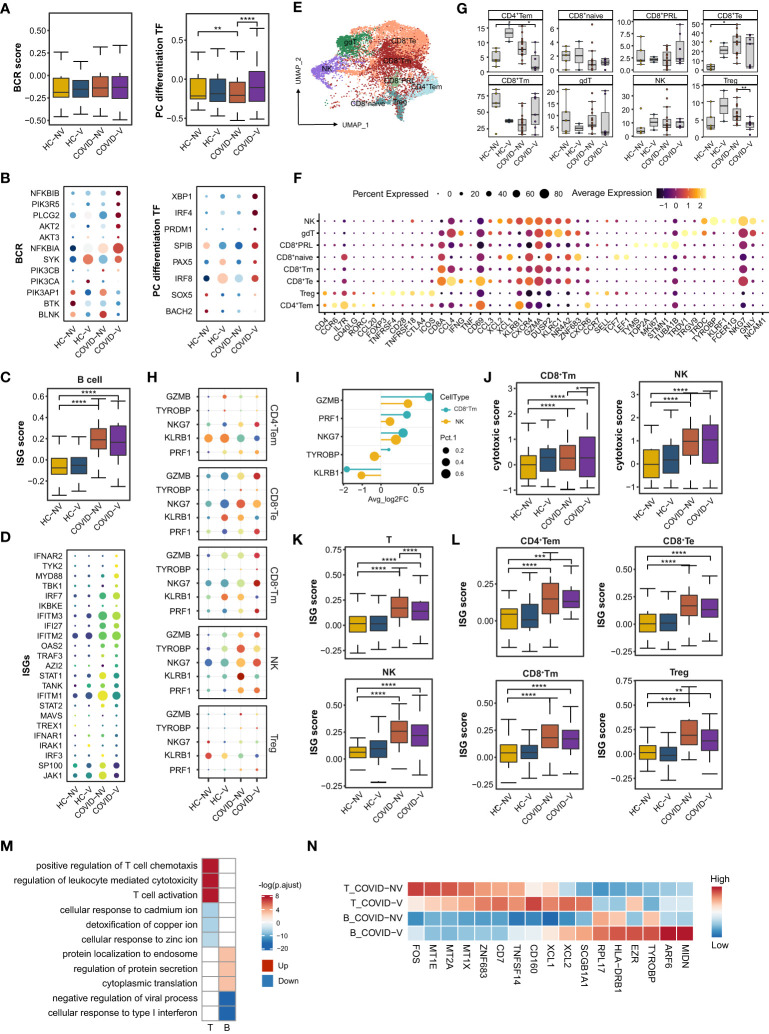
SARS-CoV-2 vaccination promotes differentiation of nasopharyngeal mucous B cells into plasma cells and the cytotoxic function of nasopharyngeal CD8^+^Tm cells. **(A)** The gene expression score of BCR signaling and transcription factors required for plasma cell differentiation in B cells from vaccinated and non-vaccinated healthy participants and patients with COVID-19. **(B)** The heatmaps of indicated genes in B cells from each vaccinated and non-vaccinated healthy participant and patients with COVID-19. **(C)** The gene expression score of ISG in B cells from vaccinated and non-vaccinated healthy participants and patients with COVID-19. **(D)** The heatmaps of selected ISGs in B cells from vaccinated and non-vaccinated healthy participants and patients with COVID-19. **(E)** UMAP representation of the eight T-cell and NK-cell types from the integrated nasopharyngeal scRNA-seq dataset. **(F)** Canonical gene expression markers for nasopharyngeal T cells and NK cells clustering. **(G)** Proportions of various nasopharyngeal T cells and NK cells from vaccinated and non-vaccinated healthy participants and patients with COVID-19. **(H)** The heatmaps of representative cytotoxic genes in various T-cell and NK-cell clusters from vaccinated and non-vaccinated healthy participants and patients with COVID-19. **(I)** Fold change of cytotoxic genes in CD8^+^Tm cells and NK cells from breakthrough infections compared with non-vaccinated patients with COVID-19. **(J)** The gene expression score of cytotoxic genes in CD8^+^Tm cells and NK cells from vaccinated and non-vaccinated healthy participants and patients with COVID-19. **(K)** The gene expression score of ISG T cells and NK cells from vaccinated and non-vaccinated healthy participants and patients with COVID-19. **(L)** The gene expression score of ISG in various nasopharyngeal T cells from vaccinated and non-vaccinated healthy participants and patients with COVID-19. **(M)** Enrichment of GO biological process terms for DEGs in nasopharyngeal T and B cells from vaccine breakthrough infections compared with non-vaccinated natural infections. **(N)** The representative DEGs enriched in signaling pathways specifically expressed in nasopharyngeal T and B cells from vaccine breakthrough infections. HC-NV, n = 6; HC-V, n = 3; COVID-NV, n = 22; COVID-V, n = 9. Data from **(A, C, G, J, K, L)** were analyzed by Mann–Whitney U-test, and data from **(G)** were analyzed by two-side Student’s t-test; *P <0.05, **P < 0.01, ***P < 0.001 and ****P < 0.0001.

B cells are recognized for generating antigen-specific antibodies to neutralize virus particles in both serum and upper respiratory tract mucous. However, we did not perform BCR repertoire sequencing integrated with transcriptome due to the sparse amount of nasopharyngeal B cells. However, we analyzed the transcription of genes involved in B-cell receptor (BCR) signaling and differentiation into antibody-secreting cells (ASCs) to evaluate the activation status and function of B cells. Some genes determining B-cell transition into ASCs were actively and universally expressed in vaccinated patients with breakthrough infection. In particular, AKT serine/threonine kinase (AKT), phospholipase C gamma (PLCγ), nuclear factor kappa B (NF-κB) and spleen associated tyrosine kinase (SYK); transcription factors paired box 5 (PAX5), interferon regulatory factor 4 (IRF4), interferon regulatory factor 8 (IRF8) and PR/SET domain 1 (PRDM1); and the corresponding gene expression score of plasma cell differentiation were significantly upregulated (**Figures 5A, B**; **S5A**). Because adaptive immunity was usually activated during the second week after infection, samples were sub-grouped into 0–3 days and over 7 days after infection (**Figures S5D, E**). Further comparison supported the rapid switching of B cell into ASCs during 0–3 days after infection, quite early stage (**Figures S5F, G**), suggestive of the promoting role for adaptive immunity activation by SARS-Cov-2 vaccination.

Similarly, nasopharyngeal B cells from vaccinated patients with COVID-19 also showed slightly attenuated ISG expression, revealed by both bulk (**Figures 5C, D**) and single-patient statistics (**Figure S5B**). The alteration of differentiation and type I response on B cells from vaccinated patients could also be observed regardless of the type of variant (**Figure S5C**). The genes involved in protein synthesis and secretion, such as eukaryotic translation elongation factor 2 (EEF2), ezrin (EZR), ADP ribosylation factor 6 (ARF6) and midnolin (MIDN), were also actively expressed on B cells (**Figures 5M, N**), indicating similar changes with epithelial cells to exert their anti-virus responses.

CD8^+^ T cells, CD4^+^ T cells, and CD4^−^CD8^−^ gamma-delta T cells constituted the nasopharyngeal mucous T lymphocytes (32) (**Figures 5E, F**). Because natural killer (NK) cells shared similar gene expression characteristics with T cells, they were distributed in the same island with CD8^+^T cells (**Figure 4A**). CD8^+^T cells accounted for the vast majority of nasopharyngeal mucous T lymphocytes. They could be further sub-clustered into naïve CD8^+^ T cells (CD8^+^T naïve), memory CD8^+^T cells (CD8^+^Tm), effector CD8^+^T cells (CD8^+^Te), and proliferative CD8^+^T cells (CD8^+^PRL) (**Figures 5E, F**). Conversely, nasopharyngeal CD4^+^T cells consisted of regulatory T cells (Treg) and effector memory CD4^+^T cells (CD4^+^Tem) (32) (**Figures 5E, F**). SARS-CoV-2 variants infection largely reduced CD8^+^Tm cells and increased CD8^+^Te cells (**Figure 5G**). As the phenotype in epithelial cells and other nasopharyngeal immune cells, the changes were impeded by inactivated SARS-CoV-2 vaccines to some degree, keeping the subset proportion similar to healthy levels (**Figure 5G**). NK and CD8^+^T cells are classically considered responsible for the specific killing of virus-infected cells. The analysis in the above subsets revealed that NK cells most highly expressed cytotoxic genes in both non-vaccinated and vaccinated patients, but CD8^+^Tm cells were mostly augmented to express cytotoxic genes by breakthrough infection (**Figures 5H, I**), indicated by either individual patient or overall samples comparison (**Figures 5J**; **S5H–J, L**). Meanwhile, the vaccinated patients showed a significant ISG reduction in all the types of nasopharyngeal T and NK subsets according to IFN scores and heatmaps of ISGs from individuals involved in the study (**Figures 5K, L**; **S5K**). These data indicated that virus-specific CD8^+^Tm cells induced by vaccination were likely formed after vaccination and re-activated and rapidly propagated during the early stage of breakthrough infection.

We further performed TCR sequence analysis and found an obvious T-cell clonal expansion for CD8^+^ Tm, CD8^+^ Te, CD8^+^ PRL, and CD4^+^ Tem cells in vaccinated patients with breakthrough infection. In detail, T-cell clonal expansion could be found in two non-vaccinated patients and three vaccinated patients infected with the alpha variant at the very early stage (3 days after PCR-proven SARS-CoV-2 infection) during infection (**Figure S5M**). We also found that vaccinated individuals with breakthrough infection were associated with larger clones (> 6) than non-vaccinated individuals with natural infection, which accounted for 20% of CD8^+^Tm, CD8^+^Te, and CD8^+^PRL cells (**Figure S5M**). This also indicated that inactivated SARS-CoV-2 vaccines could establish virus-specific TCR clone formation in nasopharyngeal mucous, which could be rapidly induced to proliferate to constrain virus spread during breakthrough infection.

A series of alterations of B and T cells indicated that vaccination would facilitate the formation of T-cell and B-cell function and state amelioration in patients with breakthrough infection by increased expression of cytotoxic genes, rapid clonal expansion, as well as upregulation of activation-related signaling pathways (T-cell activation, regulation of leukocytes mediated cytotoxicity, and positive regulation of chemotaxis) and genes (XCL1/2 and TNFSF14) according to GO analysis (**Figures 5M, N**). However, cellular responses to many types of metal ions maintained a silent state in T cells from vaccinated patients (**Figures 5M, N**), which was also recently reported as beneficial for preventing virus replication.

### The increased recruitment of macrophages and subsequent activation of lymphocytes in nasopharyngeal mucosal

**Figure 6:**
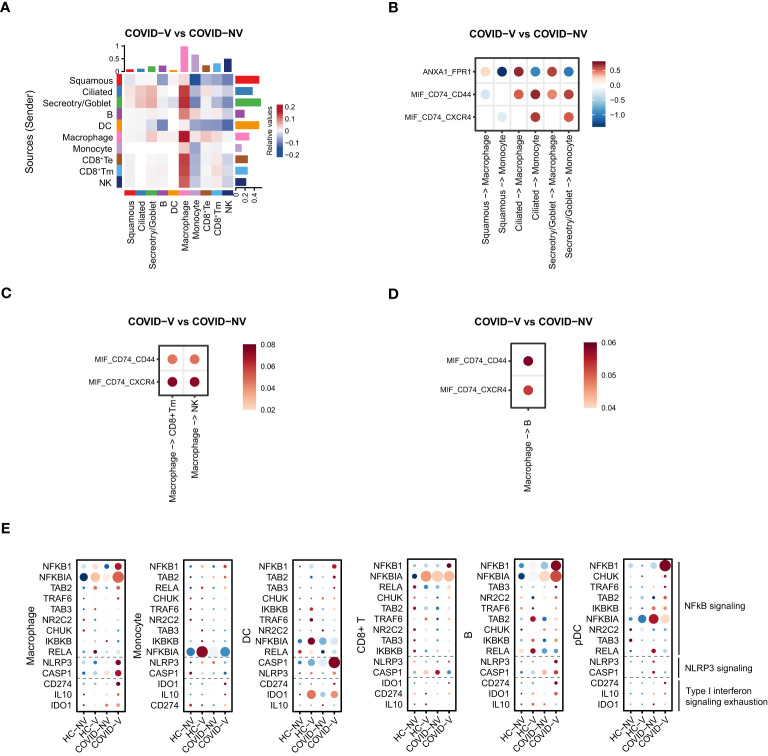
SARS-CoV-2 vaccination comprehensively elevated the interaction of nasopharyngeal macrophage with other cell populations to achieve their respective functions in vaccine breakthrough infections. **(A)** The heatmap of cell interaction between different nasopharyngeal cell types from vaccine breakthrough infections, compared with non-vaccinated patients with COVID-19. **(B)** The heatmap of cell interaction between various nasopharyngeal epithelial cell populations with macrophage/monocyte from vaccine breakthrough infections *via* indicated ligands and receptors, compared with non-vaccinated patients with COVID-19. **(C)** The heatmap of cell interaction between nasopharyngeal macrophage/monocyte with CD8^+^ Tm/NK cell from vaccine breakthrough infections *via* indicated ligands and receptors, compared with non-vaccinated patients with COVID-19. **(D)** The heatmap of cell interaction between nasopharyngeal macrophage/monocyte with B cell from vaccine breakthrough infections *via* indicated ligands and receptors, compared with non-vaccinated patients with COVID-19. **(E)** The representative NF-κB and inflammasome signaling related genes and factors involved in type I interferon exhaustion expressed in various nasopharyngeal immune cells from vaccinated and non-vaccinated healthy participants and patients with COVID-19. HC-NV, n = 6; HC-V, n = 3; COVID-NV, n = 22; COVID-V, n = 9.

We further specified the association between different cell populations. The cell-to-cell interaction analysis implied that the interaction between macrophages with three major types of epithelial cells, goblet/secretory cells, ciliated cells, and squamous cells was significantly enhanced in individuals with breakthrough infection (**Figure 6A**). This intimate interaction was complicated through CD74/CD44 or FPR1 on macrophages with macrophage migration inhibitory factor (MIF) or annexin A1 (ANXA1) on epithelial cells, respectively (**Figures 6B**, **S6A, B**), rather than the classical chemokine-chemokine receptor described in **Figure 4**. Thus, this interaction increased macrophage proportion in vaccinated patients (**Figure 4C**) and impacted the pro-inflammatory function of innate immune cells (**Figures 4D, E**). Once directly primed by epithelial cells, macrophages would further interact with CD8^+^Tm cells and B cells and promote their activation during a breakthrough infection and also recruit monocytes and macrophages *via* positive feedback (**Figure 6A**). The interaction between macrophages and B cells also relied on MIF-CD74/CD44 ligand–receptor axis (**Figures 6D**, **S6A, B**). However, another ligand–receptor pair, MIF-CD74/CXCR4, was mainly responsible for the intimate interaction between macrophages and CD8^+^ Tm cells or NK cells (**Figures 6C**, **S6A, B**). These data suggested that the overall augmentation of nasopharyngeal immune functions seemed to result from enhanced interactions between epithelial cells and macrophages in the upper respiratory tract.

Notably, the enhancement of innate immune cell function was type I IFN-independent because the ISG expression was observed to be generally inhibited in these cells. Analysis of components for innate immunity regulation showed that the NF-κB and NLRP3 signaling–related genes were upregulated in both innate and adaptive immune cells after vaccine breakthrough infection (**Figure 6D**), which replaced type I IFN to make the major contribution to orchestrate innate immunity. In addition, the general inhibition of ISG responses was likely to be explained by “IFN exhaustion” (33), which happened during multiple infections or immune challenges characterized by the apparent expression of classical exhaustion genes *CD274* (PD-L1), *IDO1*, and *IL10* (34–36) (**Figure 6E**).

## Discussion

Vaccine efficacy is determined by the successful establishment of virus-specific cellular and humoral immunity, including neutralizing antibodies, both of which have been demonstrated to reduce symptomatic COVID-19, hospitalization, severe disease, and death (4, 12–14). In particular, the virus-specific adaptive immune responses in the upper respiratory tract define the opportunity for infection and the possibility of preventing the virus from spreading into the lower respiratory tract. SARS-CoV-2–specific memory T cells and neutralizing antibodies could be detected in the nasopharyngeal swabs and saliva of healthy vaccinated individuals after immunization by mRNA vaccines (9, 37). Although the inactivated SARS-CoV-2 vaccines effectively induce virus-specific cellular and humoral immunity in peripheral blood (3, 38–41), whether they could induce virus-specific immune responses in the upper respiratory tract is not well characterized. It is important to address the impacts of vaccination on nasopharyngeal innate immunity against the subsequent SARS-CoV-2 breakthrough infection.

Because of the limited cell number of nasopharyngeal swabs, it is difficult to use classical immunological methods, such as flow cytometry and immunofluorescence to characterize nasopharyngeal immune cells. We therefore performed scRNA-seq integrated with TCR repertoires with nasopharyngeal swabs from SARS-CoV-2–inactivated vaccine–immunized subjects with breakthrough infections with different variants and non-vaccinated patients as controls. Vaccinated patients experienced more obvious TCR clonal expansion in the nasopharyngeal mucous after breakthrough infection. Among the proliferated nasopharyngeal T cells subsets, CD8^+^ Tm expressed a much higher level of cytotoxic genes during the early stage of breakthrough infection than the counterpart in non-vaccinated patients, suggesting that inactivated vaccine also successfully induced virus-specific memory T cells in the upper respiratory tract. However, NK cells were the most important contributors to killing virus-infected target cells in vaccinated and non-vaccinated patients.

Importantly, vaccination may also reshape the transcriptome of epithelial cells. The anti-virus genes expressed in squamous, goblet/secretory, and ciliated cells were significantly biased toward ROS and protein translation–related genes except for ISGs after breakthrough infection, which is significantly different from the limited anti-virus amplitude and strategy in SARS-CoV-2 natural infection. These data indicated that vaccination could remodel nasopharyngeal epithelial cells to potentially restrain ongoing virus breakthrough infection. Meanwhile, nasopharyngeal innate immune cells from vaccine breakthrough infections were activated by the epithelial cells’ recruitment to significantly increased expression of pro-inflammatory cytokines and chemokines by macrophages and monocytes and antigen-presenting relevant genes by dendritic cells. Such immune responses of nasopharyngeal innate immune cells would facilitate the rapid propagation and enhanced cytotoxic function of virus-specific T cells as well as an activation for differentiation into ASCs of virus-specific B cells induced by breakthrough infection in vaccinated patients.

Notably, the comprehensive augmentation of innate immune responses in breakthrough infection may result from the recently defined “trained immunity”, which especially explains that the innate immune cells, including myeloid cells, NK cells, and innate lymphoid cells, could adapt their functions through epigenetic reprogramming after experiencing previous pathogen-associated molecular patterns or cytokines (42, 43). This process is considered better for the novel vaccine design. On the basis of our findings, vaccination potentially reshapes nasopharyngeal innate immune responses, which could be responsible for the quick adaptive immune responses triggered by breakthrough infection. This study, to some extent, supports the notion that mucosal booster vaccination will be a good strategy against SARS-CoV-2 infection among the vaccinated population after intramuscular injection.

The IFN-I exhaustion of immune cells was common during chronic virus infection, which might be responsible for the declining transcription of ISGs in immune cells from vaccinated patients because the exhaustion molecule expression was induced in nasopharyngeal immune cells after breakthrough infection. Notably, such promotion of innate immune cells on virus-specific T cells was independent of IFN-I responses because of the general reduction of ISG expression among nasopharyngeal innate and adaptive immune cells. Future studies should address the underlying mechanisms of IFN-I exhaustion induced by vaccination in nasopharyngeal immune cells.

Collectively, we constructed the nasopharyngeal landscape of immune cells and epithelial cells and comprehensively compared the expression of pro-inflammatory cytokines, chemokines, and anti-viral–related genes from non-vaccinated and vaccinated patients with breakthrough infection. Our findings suggest that vaccination by inactivated SARS-CoV-2 vaccines potentially strengthens the innate immune barriers and establishes virus-specific memory T-cell responses, which could be quickly activated to defend against variant breakthrough infection. Thus, this study highlights the necessity of a boost *via* nasal mucous after intramuscular immunization.

## Data and materials availability

ScRNA-seq data of nasopharyngeal swabs from 6 vaccinated healthy individuals and 10 non-vaccinated patients with COVID-19 infected with WT variant were retrieved from the Genome Sequence Archive in National Genomics Data Center, Beijing Institute of Genomics (China National Center for Bioinformation), Chinese Academy of Sciences, under accession number HRA000492 that are publicly accessible at https://ngdc.cncb.ac.cn/gsa-human/browse/HRA000492.

The raw sequence data reported in this paper have been deposited in the Genome Sequence Archive in National Genomics Data Center, Beijing Institute of Genomics (China National Center for Bioinformation), Chinese Academy of Sciences, under accession number HRA003945 that are publicly accessible at https://ngdc.cncb.ac.cn/gsa-human/browse/HRA003945.

## Data availability statement

The datasets presented in this study can be found in online repositories. The names of the repository/repositories and accession number(s) can be found below: HRA000492 and HRA003945 (GSA) at https://bigd.big.ac.cn/gsa.

## Ethics statement

The studies involving human participants were reviewed and approved by Research Ethics Committee of Shenzhen Third People’s Hospital (2022003). The patients/participants provided their written informed consent to participate in this study.

## Author contributions

XH and ZZ designed the study; XH and YL did the experiments and analyzed the data; YC and FQ analyzed the scRNA-seq data; HW performed tissue sample; GX, BY, and JM provided help with experiments; DL, XL, and XT checked case history; XT help to revise the manuscript; XH and ZZ wrote the manuscript. All authors contributed to the article and approved the submitted version.
