# Supplementary material for: Breakthrough infection evokes the nasopharyngeal innate immune responses established by SARS-CoV-2–inactivated vaccine
Source: Front Immunol. 2023 Jun 29;14:1181121. doi: 10.3389/fimmu.2023.1181121 (PMC10349640; doi:10.3389/fimmu.2023.1181121)
Supplement: Supplementary file 1 [file DataSheet_1.pdf]

Table S1. Clinical characteristics of COVID-19 patients.

Patient	Age	Gender	Disease Severity	Variant	Vaccine	Time point for sample collection	Chronic disease
HC-NV1#	36	Male	Healthy		No		None
HC-NV2#	38	Female	Healthy		No		None
HC-NV3#	33	Male	Healthy		No		None
HC-NV4#	42	Female	Healthy		No		None
HC-NV5#	40	Male	Healthy		No		None
HC-NV6#	50	Male	Healthy		No		None
HC-V1#	45	Male	Healthy		Two dose, CoronaVac		None
HC-V2#	46	Male	Healthy		Two dose, CoronaVac		None
HC-V3#	37	Male	Healthy		One dose, CoronaVac		None
COVID-NV1#	42	Male	Asymptomatic	Alpha	No	D1	None
COVID-NV2#	15	Male	Asymptomatic	Alpha	No	D1	Pneumothora
COVID-NV3#	54	Male	Asymptomatic	Alpha	No	D2	Hypertension
COVID-NV4#	34	Female	Asymptomatic	Alpha	No	D3	None
COVID-NV5#	24	Female	Moderate	Delta	No	D5	None
COVID-NV6#	38	Male	Mild	Delta	No	D6	None
COVID-NV7#	42	Female	Moderate	Delta	No	D7	None
COVID-NV8#	58	Male	Severe	Delta	No	D7	None
COVID-NV9#	50	Male	Moderate	Delta	No	D7	None
COVID-NV10#	38	Male	Asymptomatic	Alpha	No	D9	Hypertension
COVID-NV11#	20	Male	Asymptomatic	Alpha	No	D7	None

COVID-NV12#	50	Male	Asymptomatic	Alpha	No	D9	Hypertension
COVID-NV13#	40	Male	Asymptomatic	WT	No	D10	None
COVIDNV14#	38	Female	Asymptomatic	WT	No	D10	None
COVID-NV15#	35	Male	Asymptomatic	WT	No	D10	None
COVID-NV16#	46	Male	Asymptomatic	WT	No	D10	None
COVID-NV17#	42	Male	Asymptomatic	WT	No	D10	None
COVID-NV18#	37	Female	Asymptomatic	WT	No	D10	None
COVID-NV19#	44	Female	Asymptomatic	WT	No	D10	None
COVID-NV20#	33	Male	Asymptomatic	WT	No	D10	None
COVID-NV21#	35	Female	Asymptomatic	WT	No	D10	None
COVID-NV22#	40	Male	Asymptomatic	WT	No	D10	None
COVID-V1#	44	Male	Asymptomatic	Alpha	Two dose, Sinopharm BIBP	D0	None
COVID-V2#	46	Male	Asymptomatic	Alpha	Two dose, Sinopharm BIBP	D1	None
COVID-V3#	49	Male	Asymptomatic	Alpha	Two dose, Sinopharm BIBP	D1	Hypertension
COVID-V4#	33	Male	Asymptomatic	Alpha	Two dose, Sinopharm BIBP	D3	None
COVID-V5#	35	Female	Asymptomatic	Alpha	One dose, CoronaVac	D5	None
COVID-V6#	48	Male	Moderate	Alpha	One dose, Sinopharm BIBP	D7	Hepatitis B
COVID-V7#	45	Male	Asymptomatic	Alpha	Two dose, Sinopharm BIBP	D8	None
COVID-V8#	44	Male	Mild	Alpha	Two dose, Sinopharm BIBP	D9	Hypertension
COVID-V9#	48	Male	Asymptomatic	Alpha	Two dose, Sinopharm BIBP	D10	None

Supplemental figure and figure legend

Supplementary figure 1

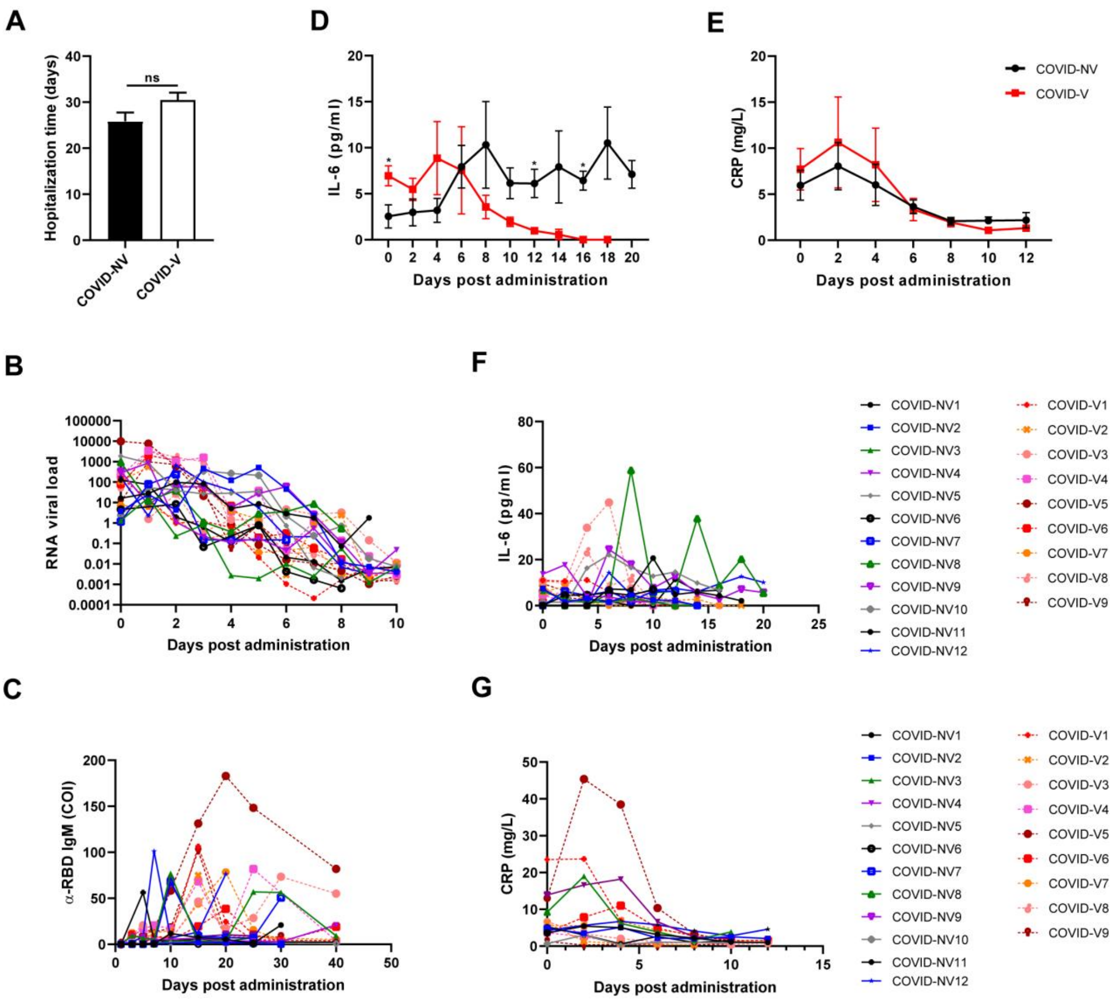

Figure S1. Clinical characteristics of vaccinated and non-vaccinated COVID-19 patients.

A. Hospitalization time of vaccinated and non-vaccinated COVID-19 patients (COVID-NV, n=12, 7 patients infected with Alpha variant and 5 patients infected with Delta variant; COVID-V, n=9, Alpha variant).

B. The RNA viral load of each COIVD-19 patient at different time points (COVID-NV, n=12; COVID-V, n=9).

C. The amount of serum anti-RBD IgM in each COIVD-19 patient at different time points (COVID-NV, n=12; COVID-V, n=9).

D. The concentration of serum IL-6 in vaccinated and non-vaccinated COVID-19 patients at different time points (COVID-NV, n=12; COVID-V, n=9).

E. The concentration of serum C reactive protein in vaccinated and non-vaccinated COVID-19

patients at different time points (COVID-NV, n=12; COVID-V, n=9).

F. The concentration of serum IL-6 in each COIVD-19 patient at different time points (COVID-NV, n=12; COVID-V, n=9).

G. The concentration of serum C reactive protein in each COIVD-19 patient at different time points (COVID-NV, n=12; COVID-V, n=9).

Data from Figure S1A were analyzed by Student's t-test, and data from Figure S1D and E were analyzed by two-way ANONVA, *P <0.05, **P < 0.01, ***P < 0.001, ns, not significant.

Supplementary figure 2

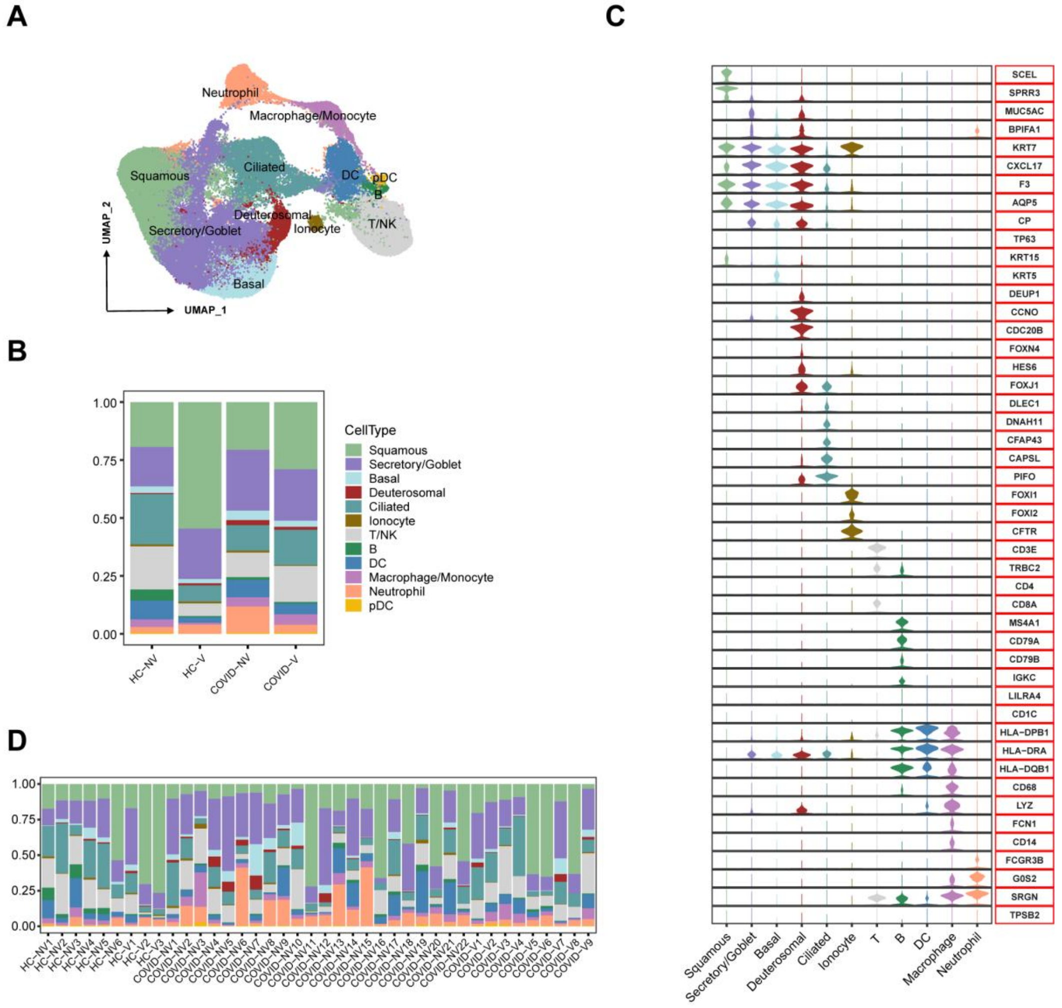

Figure S2. Overall landscape of nasopharyngeal epithelial and immune cells revealed by scRNA-seq.

A. UMAP representation of the eleven cell types from the integrated nasopharyngeal scRNA-seq dataset.

B and C. Proportions of various nasopharyngeal cell subsets from vaccinated and non-vaccinated healthy participants and COVID-19 patients.

HC-NV, n=6; HC-V, n=3; COVID-NV, n=22; COVID-V, n=9.

Supplementary figure 3

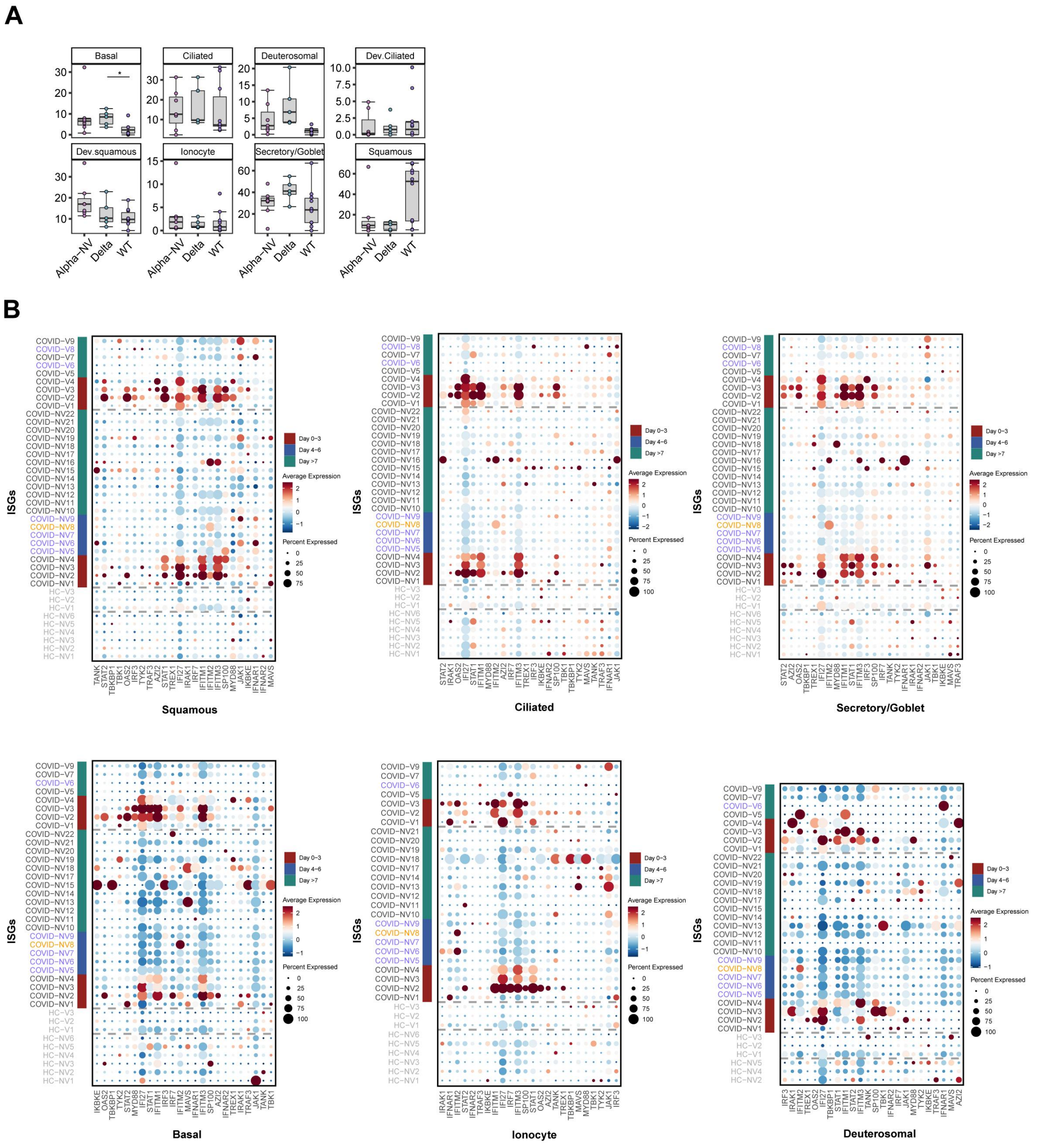

Figure S3. Supplementary to Figure 2 and Figure 3, characteristics of nasopharyngeal epithelial cells in COVID-19 patients infected with different SARS-CoV-2 variants.

A. Proportions of various nasopharyngeal epithelial cells from non-vaccinated COVID-19 patients infected with different SARS-CoV-2 variants (Alpha variant-Alpha-NV, n=7; Delta variant-Delta, n=5; wild type virus-WT, n=10).

B. The heatmaps of selected ISGs in different epithelial cells subsets from individual vaccinated and non-vaccinated healthy participants and COVID-19 patients (HC-NV, non-vaccinated healthy donor; HC-V, vaccinated healthy donor; COVID-NV, non-vaccinated COVID-19 patients; COVID-V, vaccinated patients; COVID-NV1-4, 10-12, Alpha variant; COVID-NV5-9, Delta variant;

COVID-NV13-22, wild type variant; COVID-V1-9, Alpha variant; written in gray, healthy individual; written in black, asymptomatic carrier; written in purple, mild/moderate patient; written in orange, severe patient).

Data from different groups in Figure S3A were analyzed by two-sided Student's t-test, *P <0.05, **P < 0.01, ***P < 0.001.

Supplementary Figure 4

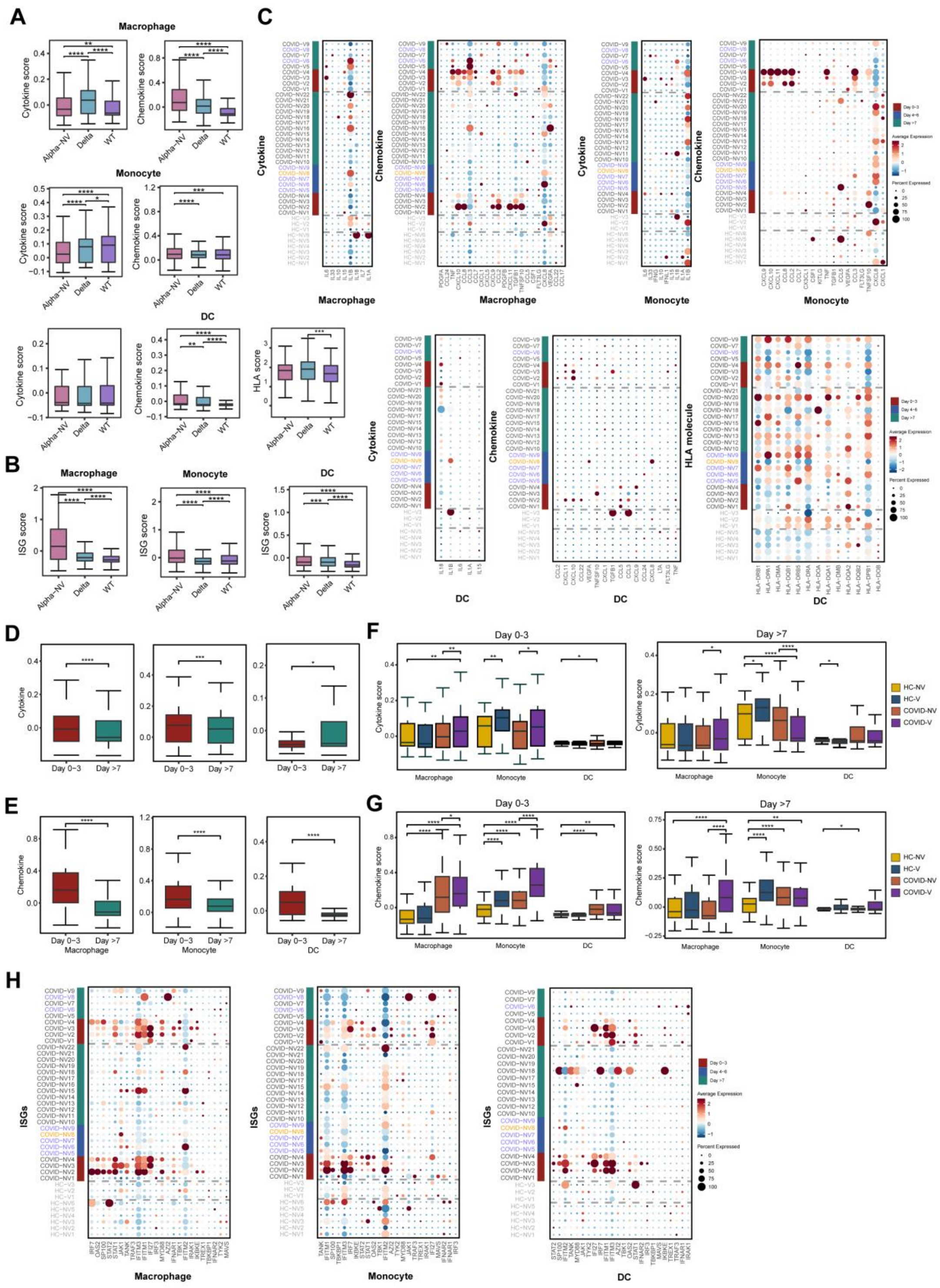

Figure S4. Supplementary to Figure 4, characteristics of nasopharyngeal innate immune cells in COVID-19 patients infected with different SARS-CoV-2 variants.

A. The gene expression score of cytokines, chemokines and MHC class II molecules in macrophages, monocytes and dentritic cells from non-vaccinated COVID-19 patients infected

with different SARS-CoV-2 variants (Alpha-NV, n=7; Delta, n=5; WT, n=10).

B. The gene expression score of ISG in macrophages, monocytes and dentritic cells from non-vaccinated COVID-19 patients infected with different SARS-CoV-2 variants.

C. The heatmaps of selected cytokines, chemokines and MHC class II molecules in macrophages, monocytes and dentritic cells from individual vaccinated and non-vaccinated healthy participants and COVID-19 patients.

D. Cytokine score of patients proven SARS-CoV-2 positive within 3 days (n=8; 4 natural infections and 4 breakthrough infections), and over 7 days (n=18, 13 natural infections and 5 breakthrough infections).

E. Chemokine score of patients proven SARS-CoV-2 positive within 3 days (n=8; 4 natural infections and 4 breakthrough infections), and over 7 days (n=18, 13 natural infections and 5 breakthrough infections).

F. The gene expression score of cytokine in macrophages, monocytes and dendritic cells from vaccinated and non-vaccinated healthy participants and COVID-19 patient during 0-3 days, or over 7 days post infection.

G. The gene expression score of chemokine in macrophages, monocytes and dendritic cells from vaccinated and non-vaccinated healthy participants and COVID-19 patient during 0-3 days, or over 7 days post infection.

H. The heatmaps of selected ISGs in macrophages, monocytes and dentritic cells from individual vaccinated and non-vaccinated healthy participants and COVID-19 patients.

Alpha-NV, n=7; Delta, n=5; WT, n=10 in Figure S4A-B. Data from different groups in Figure S4A, B, D-G were analyzed by Mann-Whitney U Test, *P <0.05, **P < 0.01, ***P < 0.001, ****P < 0.001.

Supplementary Figure 5

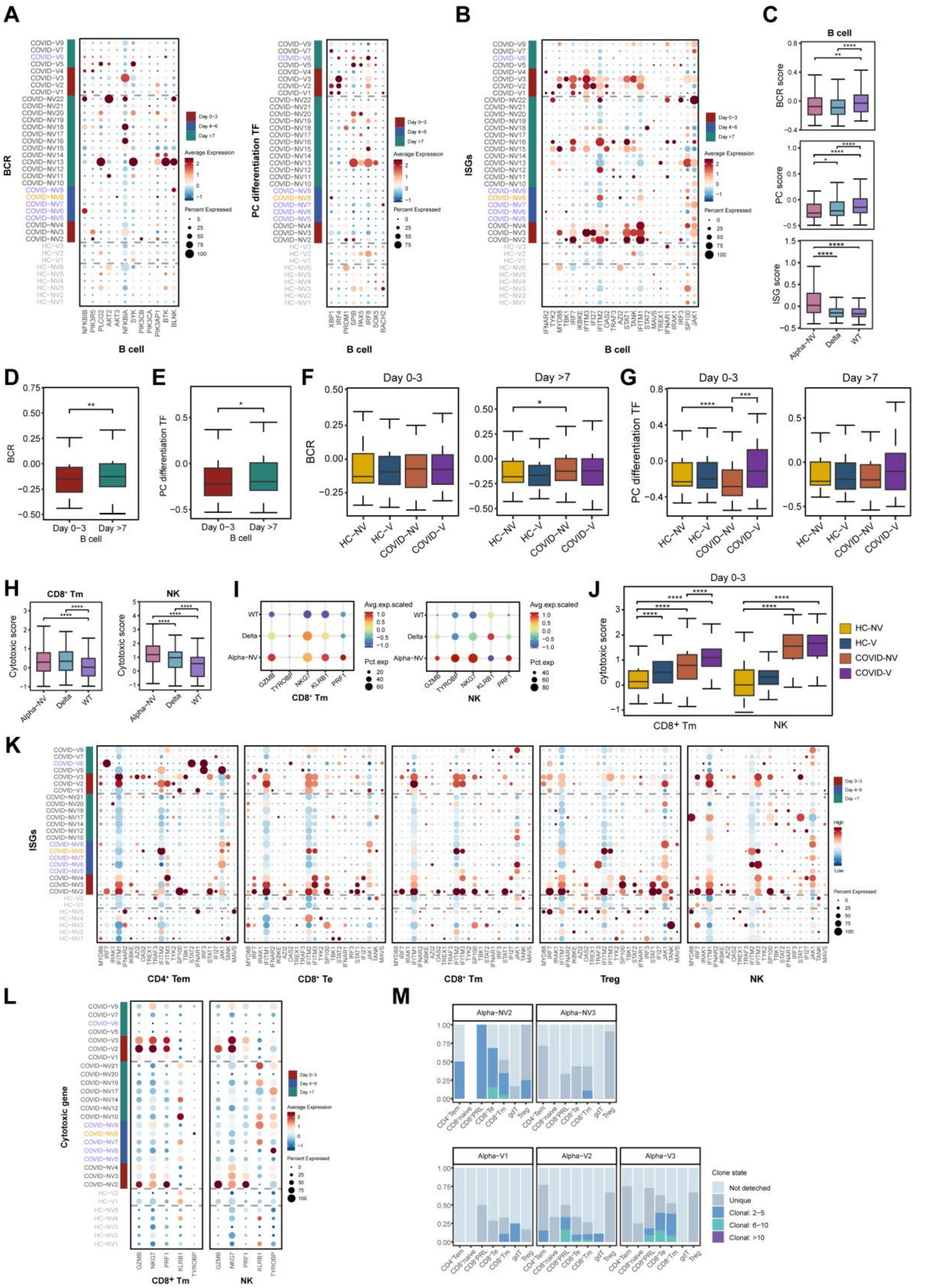

Figure S5. Supplementary to Figure 5, characteristics of nasopharyngeal B and T/NK cells in COVID-19 patients infected with different SARS-CoV-2 variants.

A. The heatmaps of selected BCR signaling related genes and transcription factors required for

plasma cell differentiation in B cells from individual vaccinated and non-vaccinated healthy participants and COVID-19 patients.

B. The heatmaps of ISGs in B cells from individual vaccinated and non-vaccinated healthy participants and COVID-19 patients.

C. The gene expression score of BCR signaling, transcription factors required for plasma cell differentiation and ISG in B cells from non-vaccinated COVID-19 patients infected with different SARS-CoV-2 variants.

D. BCR score of patients proven SARS-CoV-2 positive within 3 days (n=8; 4 natural infections and 4 breakthrough infections), and over 7 days (n=18, 13 natural infections and 5 breakthrough infections).

E. Transcription factors score for plasma cell differentiation of patients proven SARS-CoV-2 positive within 3 days (n=8; 4 natural infections and 4 breakthrough infections), and over 7 days (n=18, 13 natural infections and 5 breakthrough infections).

F. The gene expression score of BCR signaling in B cells from vaccinated and non-vaccinated healthy participants and COVID-19 patients during 0-3 days or over 7 days post infection.

G. The gene expression score of transcription factors required for plasma cell differentiation in B cells from vaccinated and non-vaccinated healthy participants and COVID-19 patients during 0-3 days or over 7 days post infection.

H. The gene expression score of cytotoxic in $CD8^{+}$Tm and NK cells from non-vaccinated COVID-19 patients infected with different SARS-CoV-2 variants.

I. The heatmaps of representative cytotoxic genes in $CD8^{+}$Tm and NK cells from non-vaccinated COVID-19 patients infected with different SARS-CoV-2 variants.

J. The gene expression score of cytotoxic in $CD8^{+}$Tm and NK cells from vaccinated and non-vaccinated healthy participants and COVID-19 patients during 0-3 days or over 7 days post infection.

K. The heatmaps of ISGs in various T cell subsets and NK cells from individual vaccinated and non-vaccinated healthy participants and COVID-19 patients.

L. The heatmaps of cytotoxic genes in $CD8^{+}$Tm and NK cells from individual vaccinated and non-vaccinated healthy participants and COVID-19 patients.

M. Clonal expansion of various T cell subsets from individual vaccinated and non-vaccinated

COVID-19 patients.

Alpha-NV, n=7; Delta, n=5; WT, n=10 in Figure S5C, H. Data from different groups in Figure S5C-H and J were analyzed by Mann-Whitney U Test, *P <0.05, **P < 0.01, ***P < 0.001, ****P < 0.001.

Supplementary Figure 6

A

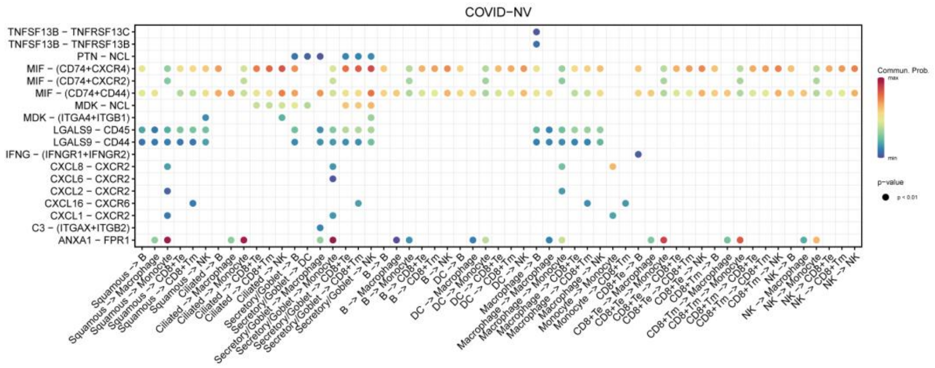

B

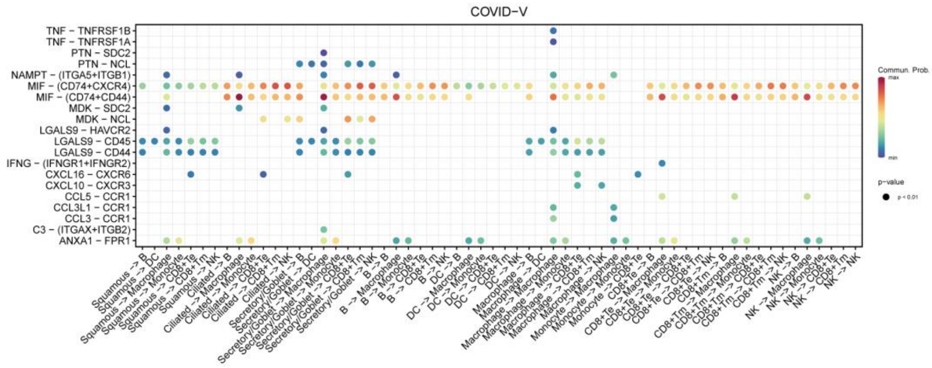

Figure S6. Supplementary to Figure 6, SARS-CoV-2 vaccination comprehensively elevated the interaction of nasopharyngeal macrophage with other cell populations.

A. The ligand-receptor interaction map of different nasopharyngeal cells from non-vaccinated COVID-19 patients.

B. The ligand-receptor interaction map of different nasopharyngeal cells from vaccinated COVID-19 patients.

COVID-NV, n=22; COVID-V, n=9 in Figure S6.
